# External validation as the critical step for predictive biomarkers

**DOI:** 10.1093/ijnp/pyag036

**Published:** 2026-06-25

**Authors:** Pál Czobor, Brigitta Kakuszi, Máté Fullajtár, István Bitter

**Affiliations:** Department of Psychiatry and Psychotherapy, Semmelweis University, Budapest, Hungary; Department of Psychiatry and Psychotherapy, Semmelweis University, Budapest, Hungary; Department of Psychiatry and Psychotherapy, Semmelweis University, Budapest, Hungary; Department of Psychiatry and Psychotherapy, Semmelweis University, Budapest, Hungary

We thank Du^1^ for the thoughtful commentary regarding our recently published study on high-density electroencephalography (EEG) in patients with schizophrenia treated with cariprazine.^2^ We appreciate the recognition of the findings as novel and potentially important.

Overall, the commentary suggests that the conclusions regarding the predictive utility of baseline EEG gamma activity would benefit from more cautious framing. Several specific issues were raised, and we welcome the opportunity to reflect further on the interpretation and implications of our findings.

Two concerns were raised regarding the interpretation of predictive biomarkers, both referencing prior meta-analytic work. First, the commentary cites a systematic review and exploratory meta-analysis of patient-control studies in schizophrenia,^3^ which reported mixed directions of effect and a non-significant pooled case–control difference with substantial heterogeneity. While we agree with Du’s conclusion that “baseline gamma may well represent a promising candidate signal, but not yet a mature response-stratification biomarker,” we note that the cited meta-analysis primarily addressed the utility of gamma activity measures as diagnostic biomarkers. Therefore, its relevance to treatment-response prediction is limited.

Second, the commentary refers to the systematic review by Meehan et al.^4^ which reported that 94.5% of psychiatric prediction models were at high risk of bias. Although this is an important concern for the field overall, the applicability of these findings to the present study is also limited. Only approximately one-third of the reviewed studies concerned psychosis, and the majority focused on diagnostic or prognostic biomarkers related to illness onset or course. In addition, most of the included prediction models relied predominantly on sociodemographic and clinical predictors rather than biological measures.

The commentary also raised several methodological questions regarding our analyses. One question concerned whether the findings would persist if the Positive and Negative Syndrome Scale (PANSS) change were analyzed as a continuous rather than dichotomized outcome. To address this issue, we conducted this analysis using continuous change-from-baseline scores. The association remained highly statistically significant with a large effect size, including the gamma-2 range (*R*^2^ = 0.48, *P* < .005), where the decrease in cross-validation performance from the validation to the test sample was most pronounced.

Another question concerned whether optimism-corrected performance estimates and calibration measures were examined. Regarding calibration, our primary interest was not absolute probability calibration per se, but rather discriminative and probabilistic classification performance since a model may exhibit excellent calibration while remaining clinically uninformative in terms of discrimination. Nevertheless, we additionally examined the scaled Brier score, which quantifies improvement in probabilistic prediction performance relative to a non-informative baseline model defined by the marginal event rate.^5^^,^^6^ The value was 20%, which may be considered clinically meaningful and was consistent with the leave-one-out-cross-validation (LOOCV)-based estimates.

With respect to optimism correction, we note that LOOCV is itself an inherently optimism-corrected procedure because it evaluates out-of-sample rather than resubstitution performance. If the commentary refers specifically to bootstrap-based optimism correction methods, we would note that although such approaches are widely discussed for internal validation, their practical utility remains unresolved. Current evidence suggests that bootstrap-based correction methods may sometimes underperform relative to cross-validation approaches, yielding estimates that are either overly optimistic or excessively pessimistic depending on the context. Indeed, a recent simulation study by Zhang et al.^7^ concluded that “given that cross-validation is more robust and computationally less costly to implement than the bootstrap-based methods, it should be recommended as a preferred method for internal validation of prediction models.”

Regarding the broader framing of baseline gamma activity as an exploratory predictive signal, we would emphasize that the exploratory nature of the work was explicitly acknowledged throughout the manuscript. The opening sentence of the discussion refers to the study as an “exploratory high-density EEG study,” and the manuscript consistently acknowledges the modest sample size and the need for replication in larger and independent cohorts to establish robustness and generalizability. Thus, we agree that the limited number of responders represents a constraint on the interpretation of the findings. However, we also note that the predictive performance was not restricted to the responder subgroup alone. Classification performance for non-responders was of comparable magnitude (>70% under LOOCV). This suggests that the observed effects were not solely driven by the relatively small responder subgroup, which is consistent with the results of the analyses of the continuous responses based on the full sample of subjects. Nonetheless, replication in larger independent cohorts remains essential. Overall, although several aspects of the findings are consistent with prior pharmaco-EEG literature, we believe that the central issue is ultimately not the choice among alternative internal validation procedures but rather successful replication in independent datasets.

Finally, we are grateful to Du for the opportunity to further discuss these important methodological issues in a professional forum. Internal validation and optimism-correction procedures clearly play an important role in the development of predictive models. At the same time, the growing number and complexity of such approaches may sometimes create the impression that methodological refinement alone can substitute for independent confirmation. In our view, the ultimate test of the robustness and clinical relevance of predictive biomarkers remains successful replication and external validation in independent datasets. Accordingly, we believe that future efforts should place increasing emphasis not only on internal validation strategies but also on study designs that facilitate reproducibility and independent confirmation of findings.
